# Nordihydroguaiaretic Acid Disrupts the Antioxidant Ability of *Helicobacter pylori* through the Repression of SodB Activity *In Vitro*

**DOI:** 10.1155/2015/734548

**Published:** 2015-04-06

**Authors:** Hitoshi Tsugawa, Hideki Mori, Juntaro Matsuzaki, Tatsuhiro Masaoka, Tasuku Hirayama, Hideko Nagasawa, Yasubumi Sakakibara, Makoto Suematsu, Hidekazu Suzuki

**Affiliations:** ^1^Department of Biochemistry & Integrative Medical Biology, School of Medicine, Keio University, 35 Shinanomachi, Shinjuku-ku, Tokyo 160-8582, Japan; ^2^Division of Gastroenterology and Hepatology, Department of Internal Medicine, School of Medicine, Keio University, 35 Shinanomachi, Shinjuku-ku, Tokyo 160-8582, Japan; ^3^Laboratory of Pharmaceutical & Medicinal Chemistry, Gifu Pharmaceutical University, 1-25-4 Daigaku-nishi, Gifu 501-1196, Japan; ^4^Biosciences and Informatics, Keio University, 3-14-1 Hiyoshi, Yokohama 223-8522, Japan

## Abstract

Iron-cofactored superoxide dismutase (SodB) of *Helicobacter pylori* plays an indispensable role in the bacterium's colonization of the stomach. Previously, we demonstrated that FecA1, a Fe^3+^-dicitrate transporter homolog, contributes to SodB activation by supplying ferrous iron (Fe^2+^) to SodB, and *fecA1*-deletion mutant strains have reduced gastric mucosal-colonization ability in Mongolian gerbils, suggesting that FecA1 is a possible target for the development of a novel eradication therapy. This study aimed to identify novel FecA1-binding compounds *in silico* and then examined the effect of a predicted FecA1-binding compound on *H. pylori* SodB activity *in vitro*. Specifically, we demonstrated that nordihydroguaiaretic acid (NDGA) is a predicted FecA1-binding compound. NDGA reduced intracellular Fe^2+^ levels in *H. pylori* and reduced SodB activity. Additionally, NDGA increased H_2_O_2_ sensitivity of *H. pylori* and increased the metronidazole (Mtz) sensitivity. The present study demonstrated that NDGA repressed SodB activity associated with the gastric mucosal-colonization via inhibition of intracellular Fe^2+^ uptake by FecA1, suggesting that NDGA might be effective for the development of a novel eradication therapy.

## 1. Introduction

*Helicobacter pylori* infection is one of the most common infectious diseases worldwide. Chronic infection of* H. pylori* plays a causative role in gastritis, peptic ulcer, and gastric carcinomas [1]. Excessive reactive oxygen species (ROS) are generated by neutrophils and macrophages in* H. pylori* colonized human stomachs [2]. Although the generation of ROS is an important host immune response against persistent pathogens,* H. pylori* counteracts oxidative stress using a variety of enzymes to establish a chronic infection [3, 4]. Therefore, persistent and excessive ROS induces oxidative stress injuries to the gastric mucosa.

The* H. pylori* genome encodes iron-cofactored superoxide dismutase (SodB) [5, 6]. Mutants with a deletion of* sodB* show a reduced gastric mucosal-colonization ability in mice, suggesting that SodB plays an indispensable role in the colonization of host gastric mucosa owing to its defensive role against excessive ROS [7]. Ferrous ion (Fe^2+^) is required for the activation of SodB. We recently demonstrated that the deletion mutant of the* fecA1 *gene, a Fe^3+^-dicitrate transporter homolog, shows reduced SodB activity [8]. Our previous findings suggested that the Fe^2+^-supply system associated with SodB activation proceeds as follows: Fe^3+^-dicitrate transport is enhanced by FecA1, and then intracellular ferric ion (Fe^3+^) is reduced to Fe^2+^ by Fe^3+^-reductase (ribBA), providing Fe^2+^ to SodB [8, 9]. In addition,* fecA1*-deletion mutant strains show reduced gastric mucosal-colonization ability in Mongolian gerbils [8]. These results indicated that* fecA1-*deletion mutant strains were eradicated by excessive ROS in host gastric mucosa via disruption of SodB activity. In fact, FecA1 associated with SodB activation is an important determinant of the establishment of chronic infections.

Recently, we demonstrated that SodB overexpression is caused by amino acid mutations of ferric uptake regulator (Fur), which is associated with the development of metronidazole (Mtz) resistance [10]. The deletion of* fecA1* in Mtz-resistant strains (KS0048 and KS0145) results in increased Mtz sensitivity, suggesting that FecA1 is also associated with the development of Mtz resistance [8].

Our previous results indicated that FecA1 is a possible target for the development of a selective* H. pylori* eradication therapy mediated by excessive ROS accumulation in the human stomach and for avoiding the development of Mtz resistance associated with SodB overexpression. In the present study, we identified a compound that demonstrated an inhibitory effect on SodB activity via the inhibition of FecA1.

## 2. Materials and Methods

### 2.1. Bacterial Strains and Culture Conditions

*H. pylori* strains ATCC700392, KS0048, and KS0145 were used in this study. The KS strains were clinically isolated and maintained at −80°C in Brucella broth (Becton–Dickinson, Franklin Lakes, NJ, USA) containing 25% (vol/vol) glycerol. The KS0048 and KS0145 strains were used as the Mtz-resistant strains with SodB overexpression owing to Fur amino acid mutations. ATCC700392 pHel3::*sodB* strain was used as the SodB-overexpressing strain of* H. pylori* ATCC700392, and ATCC700392 pHel3* ctrl* strain was used as the control strain of ATCC700392 pHel3::*sodB* strain [10]. It was confirmed that the SodB activity in ATCC700392 pHel3::*sodB* was higher as compared with that of ATCC700392 pHel3* ctrl* strain [10]. ATCC700392Δ*fecA1*, KS0048Δ*fecA1*, and KS0145Δ*fecA1* strains were used as* fecA1*-deletion mutant strains of* H. pylori* ATCC700392, KS0048, and KS0145, respectively [8]. It was confirmed that the SodB activities in* fecA1*-deletion mutant strains were significantly decreased as compared with wild-type strains [8]. These strains were maintained at −80°C in Brucella broth (Becton–Dickinson, Franklin Lakes, NJ, USA) containing 25% (vol/vol) glycerol. The bacteria were cultured on Brucella agar containing 7% sheep blood and 7% fetal bovine serum for 2 days at 37°C under microaerobic conditions maintained with AnaeroPack MicroAero (Mitsubishi Gas, Tokyo, Japan).

### 2.2. *In Silico* Screening of Chemical Compounds with Binding Affinity to FecA1

The FecA1 amino acid sequence of* H. pylori* (UniProt ID: O25395) was inserted into the COPICAT (Comprehensive Predictor of Interactions between Chemical Compounds and Target Proteins) program, which is an* in silico* screening system to predict the comprehensive interactions between target proteins and chemical compounds [11]. A chemical compound that is likely to interact with FecA1 was predicted.

### 2.3. Measurement of Intracellular Fe^2+^ Levels

The bacteria normalized to an OD_600_ of 0.5 were incubated with or without nordihydroguaiaretic acid (NDGA) for 3 hr. The bacteria were washed three times with HBSS and then 10 *μ*M RhoNox-1 (from 1 mM stock solution in DMSO) was added [12]. After incubation for 1 hr at 37°C, the bacteria were washed with HBSS and then normalized to an OD_600_ of 0.1. Fluorescence intensity was measured using 560 nm excitation and 595 nm emission.

### 2.4. Measurement of SOD Activity

The bacteria normalized to an OD_600_ of 0.5 were incubated with or without NDGA for 3 hr. The bacteria were centrifuged, were washed three times with PBS, and then were sonicated (1.5 min at 25% power). The bacterial lysates were centrifuged, and the SOD activity was measured using the SOD Assay Kit (Dojindo, Kumamoto, Japan) following the manufacturer's guidelines [8]. Protein concentrations of the bacterial lysates were measured by BCA assays (Thermo Scientific, Rockford, IL, USA).

### 2.5. Measurement of the MICs of Mtz

The bacteria normalized to an OD_600_ of 0.5 were incubated with or without 50 *μ*M NDGA for 3 hr. The bacteria (at an OD_600_ of 0.1) were inoculated on an agar plate containing Mtz in serial twofold dilutions (0.5–128 *μ*g/mL). All plates were incubated at 37°C under microaerobic conditions, and the minimum inhibitory concentration (MIC) values were determined [8].

### 2.6. Disk Assays for H_2_O_2_ Susceptibility

The bacteria normalized to an OD_600_ of 0.5 were incubated with or without 50 *μ*M NDGA for 3 hr. The bacteria were centrifuged and washed three times with PBS. The bacteria, normalized to an OD_600_ of 0.1, were plated for confluent growth on Nissui Helicobacter agar (Nissui, Tokyo, Japan). Sterile 5 mm disks saturated with 10 *μ*L of 5 M hydrogen peroxide (H_2_O_2_) were placed on the plates. After 3 days, the zone of inhibition around the disks was measured [8].

### 2.7. Statistical Analysis

All values were expressed as means ± SD. The statistical significance of differences between three or more groups was evaluated using the Tukey test. Differences were considered to be significant for values of *P* < 0.05.

## 3. Results

### 3.1. Screening of Chemical Compounds with Binding Affinity to FecA1 of* H. pylori*

**Figure 1:**
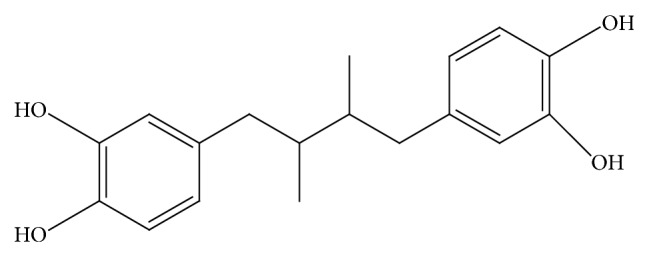
Chemical structure of nordihydroguaiaretic acid (NDGA). Other name of NDGA is Masoprocol. Chemical structural formula of NDGA was described using the ChemBioDraw13.0.

To search for chemical compounds with binding affinity to FecA1 of* H. pylori*, we used the COPICAT web-based software system [11]. Using the FecA1 protein (UniProt ID: O25395) as the COPICAT input, nordihydroguaiaretic acid (NDGA), sevoflurane, and enflurane were a predicted FecA1-binding compound. Considering the clinical application and toxicity of these compounds, sevoflurane and enflurane were excluded. We focused on the effect of NDGA on the intracellular Fe^2+^ levels and SodB activity in* H. pylori* (Figure 1).

### 3.2. Effect of NDGA on Intracellular Fe^2+^ Levels and SodB Activity in* H. pylori*

**Figure 2:**
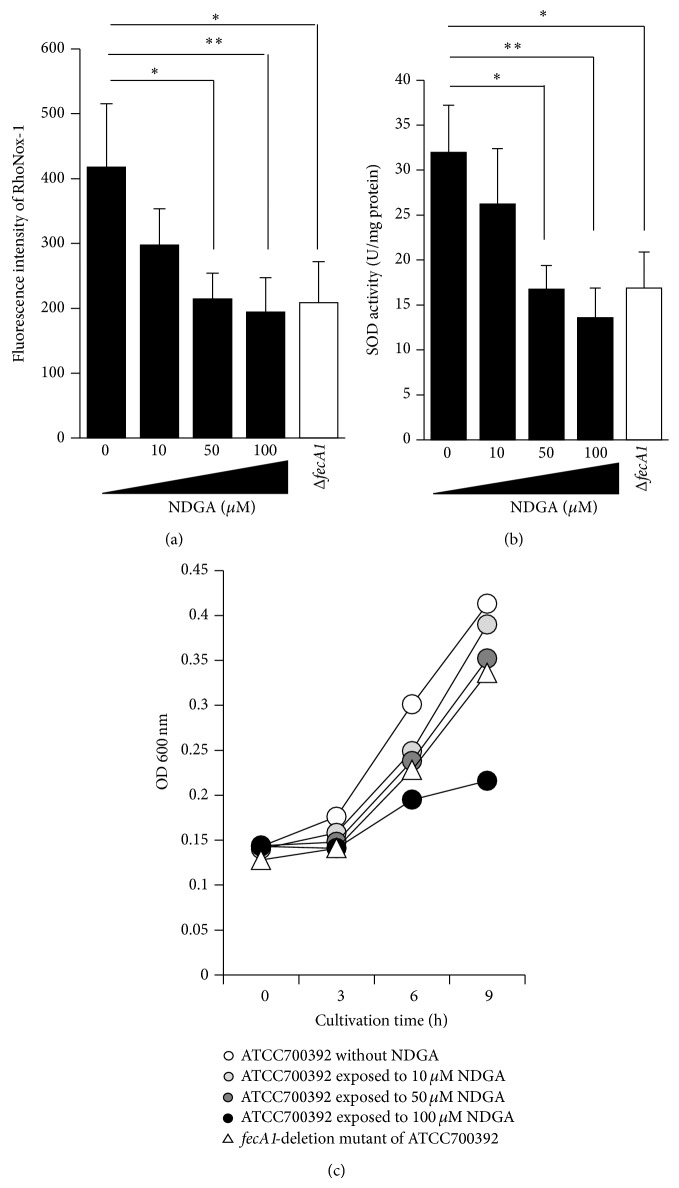
Effect of NDGA on intracellular Fe^2+^ levels, SOD activity, and bacterial growth. (a) Intracellular Fe^2+^ levels in ATCC700392 exposed to NDGA and* fecA1*-deletion mutant of ATCC700392 (Δ*fecA1*) were measured by the method described under Materials and Methods. Results are means ± SD of three independent assays. ^*^ *P* < 0.05; ^**^ *P* < 0.01 (Tukey test). (b) The SodB activities in ATCC700392 exposed to NDGA and* fecA1*-deletion mutant of ATCC700392 (Δ*fecA1*) were measured by the method described under Materials and Methods. Results are means ± SD of three independent assays. ^*^ *P* < 0.05; ^**^ *P* < 0.01 (Tukey test). (c) The growth rates of ATCC700392 without NDGA (white circle), ATCC700392 exposed to 10 *μ*M NDGA (gray circle), ATCC700392 exposed to 50 *μ*M NDGA (deep-gray circle), ATCC700392 exposed to 100 *μ*M NDGA (black circle), and* fecA1*-deletion mutant of ATCC700392 (white triangle) were measured by measuring optical density (OD 600 nm).

We examined the effect of NDGA on intracellular Fe^2+^ levels using a turn-on fluorescent probe, RhoNox-1, for the selective detection of Fe^2+^ [12]. The intracellular Fe^2+^ levels were significantly lower in NDGA-treated strains than in control strains, and this effect was dose-dependent (Figure 2(a)). Previously, we demonstrated that* fecA1*-deletion mutant strains show lower SodB activity than the wild-type strain [8]. Therefore, we investigated the effect of NDGA on SodB activity. The SodB activity was significantly decreased after exposure to NDGA in a dose-dependent manner (Figure 2(b)). Treatment with 50 *μ*M NDGA decreased the SodB activity to the same level observed in the* fecA1*-deletion mutant strains (Δ*fecA1*) (Figure 2(b)). To evaluate the effect of NDGA on the bacterial viability, we estimated growth rates after NDGA exposure by measuring optical density (OD_600_ nm). Although the bacterial growth was suppressed after exposure to 100 *μ*M NDGA, it was hardly influenced by exposure to 10 and 50 *μ*M NDGA (Figure 2(c)). Similarly, deletion of the* fecA1 *gene had little influence on the bacterial growth (Figure 2(c)). These results suggested that 50 *μ*M NDGA was a potent selective inhibitor of SodB activity in* H. pylori* via the repression of intracellular Fe^2+^ levels, without bacteriostatic activity.

**Figure 3:**
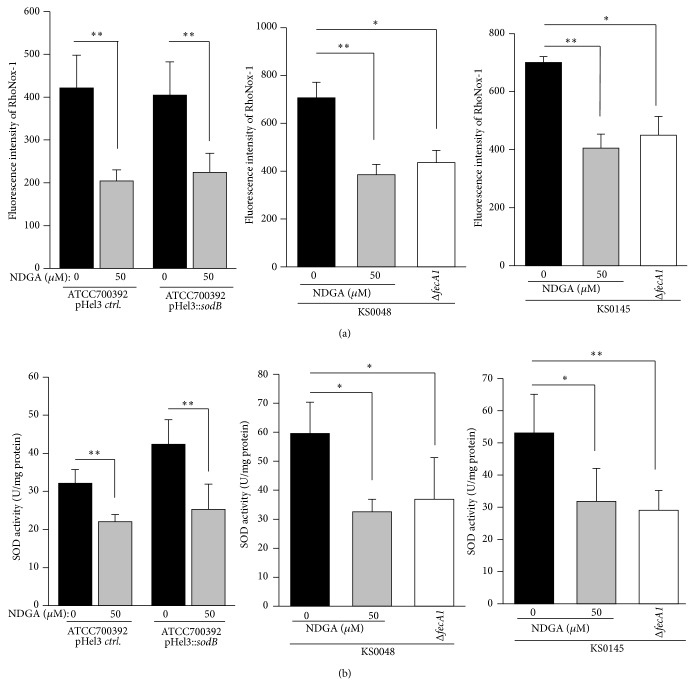
The intracellular Fe^2+^ levels and the SOD activities of Mtz-resistant strains under exposure to 50 *μ*M NDGA. (a) The intracellular Fe^2+^ levels of control strain (ATCC700392 pHel3* ctrl*.), SodB-overexpressing strain (ATCC700392 pHel3::*sodB*), KS0048,* fecA1*-deletion mutant of KS0048 (KS0048Δ*fecA1*), KS0145, and* fecA1*-deletion mutant of KS0145 (KS0145Δ*fecA1*) were measured by the method described under Materials and Methods. Results are means ± SD of three independent assays. ^*^ *P* < 0.05; ^**^ *P* < 0.01 (Tukey test). (b) The SOD activities of control strain (ATCC700392 pHel3* ctrl*.), SodB-overexpressing strain (ATCC700392 pHel3::*sodB*), KS0048,* fecA1*-deletion mutant of KS0048 (KS0048Δ*fecA1*), KS0145, and* fecA1*-deletion mutant of KS0145 (KS0145Δ*fecA1*) were measured by the method described under Materials and Methods. Results are means ± SD of three independent assays. ^*^ *P* < 0.05; ^**^ *P* < 0.01 (Tukey test).

Recently, we demonstrated that SodB activities of Mtz-resistant strains (KS0048 and KS0145) increase due to Fur amino acid mutations [10]. Therefore, we investigated whether NDGA repressed the enhanced SodB activities of KS0048 and KS0145. The intracellular Fe^2+^ levels of the SodB-overexpressing strain (ATCC700392 pHel3::*sodB*) as well as that of the control strain (ATCC700392 pHel3* ctrl*) decreased significantly for exposure to 50 *μ*M NDGA (Figure 3(a)). Similarly, the intracellular Fe^2+^ levels of KS0048 and KS0145 decreased significantly to levels comparable to the* fecA1*-deletion mutant strains (KS0048Δ*fecA1* and KS0145Δ*fecA1*, resp.) (Figure 3(a)). The SodB activities of these strains decreased significantly to the same levels as those of* fecA1*-deletion mutant strains (KS0048Δ*fecA1* and KS0145Δ*fecA1*, resp.) (Figure 3(b)). These results suggested that NDGA also repressed the enhanced SodB activities of KS0048 and KS0145.

### 3.3. Effect of NDGA on Mtz Susceptibility of* H. pylori*

Previously, we demonstrated that* fecA1*-deletion mutants of KS0048 and KS0145 have decreased MICs of Mtz [8]. Therefore, we examined whether Mtz resistance of KS0048 and KS0145 was reversed by NDGA treatment. The MICs of Mtz for KS0048 and KS0145 decreased from 32 to 8 and from 128 to 16 *μ*g/mL, respectively (Table 1). In particular, the Mtz resistance of ATCC700392 pHel3::*sodB* was completely reversed by treatment with 50 *μ*M NDGA (MIC < 8 *μ*g/mL) (Table 1).

### 3.4. Effect of NDGA on* H. pylori* H_2_O_2_ Sensitivity

**Figure 4:**
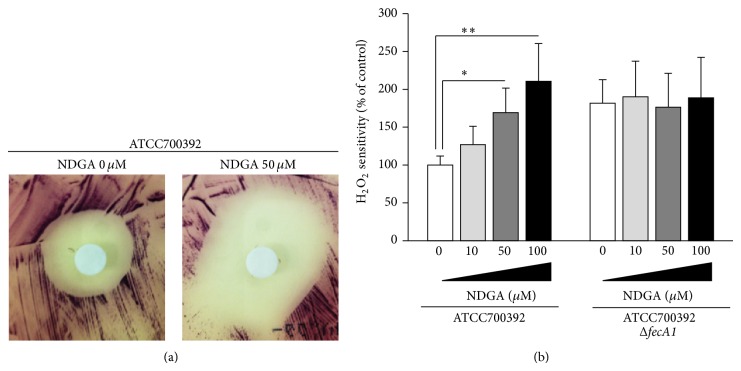
Effect of NDGA on H_2_O_2_ sensitivity. (a) Representative disk assays for H_2_O_2_ sensitivity of ATCC700392 exposed to 50 *μ*M NDGA. (b) The H_2_O_2 _sensitivity of ATCC700392 and* fecA1*-deletion mutant of ATCC700392 (ATCC700392Δ*fecA1*) under exposure to NDGA was measured by the method described under Materials and Methods. Results are means ± SD of three independent assays. ^*^ *P* < 0.05; ^**^ *P* < 0.01 (Tukey test).

**Figure 5:**
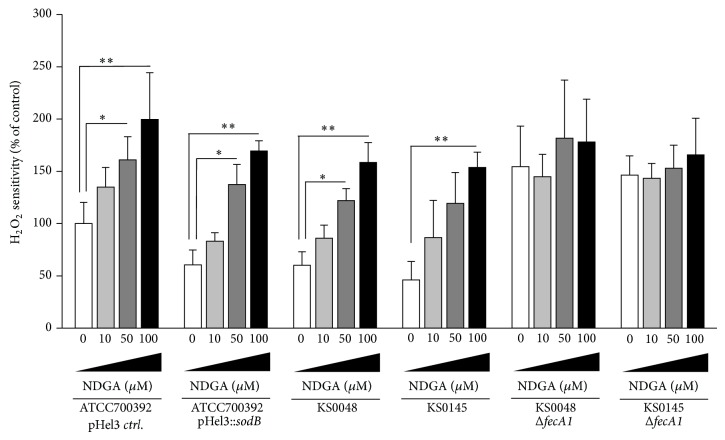
The H_2_O_2_ sensitivity of Mtz-resistant strains under exposure to NDGA. The H_2_O_2 _sensitivities of control strain (ATCC700392 pHel3* ctrl*.), SodB-overexpressing strain (ATCC700392 pHel3::*sodB*), KS0048, KS0145,* fecA1*-deletion mutant of KS0048 (KS0048Δ*fecA1*), and* fecA1*-deletion mutant of KS0145 (KS0145Δ*fecA1*) were measured by the method described under Materials and Methods. Results are means ± SD of three independent assays. ^*^ *P* < 0.05; ^**^ *P* < 0.01 (Tukey test).

To investigate whether NDGA repressed the antioxidant ability of* H. pylori*, we examined the H_2_O_2_ sensitivity of* H. pylori* by disk assays. The H_2_O_2_ sensitivity of* H. pylori* was significantly increased by NDGA treatment in a dose-dependent manner (Figures 4(a) and 4(b)). On the other hand, enhanced H_2_O_2_ sensitivity of* fecA1*-deletion mutant strains (ATCC700392Δ*fecA1*) was not affected by NDGA treatment (Figure 4(b)). Similarly, the H_2_O_2_ sensitivity of SodB-overexpressing strains (ATCC700392 pHel3::*sodB*, KS0048, and KS0145) was significantly increased after exposure to NDGA in a dose-dependent manner (Figure 5). Enhanced H_2_O_2_ sensitivity by deletion of* fecA1* genes (KS0048Δ*fecA1* and KS0145Δ*fecA1*) was not affected by NDGA treatment (Figure 5). From these results, it was thought that NDGA disrupted the SodB-dependent antioxidant ability of* H. pylori* via FecA1 inhibition.

## 4. Discussion

Previously, we demonstrated that* fecA1*-deletion mutant strains show reduced host colonization owing to the inactivation of SodB, suggesting that* H. pylori* was eradicated by excessive ROS mediated by host immune responses [8]. In this* in vitro* study, we showed that NDGA treatment inhibited the SodB activity and increased the H_2_O_2_ sensitivity to the same levels observed in* fecA1*-deletion mutant strains. Therefore, it was expected that NDGA might reduce the host-colonization ability of* H. pylori*.

Although the growth of* H. pylori* was suppressed under 100 *μ*M NDGA exposure, it was minimally influenced under 50 *μ*M NDGA, suggesting that high concentrations of NDGA showed bacteriostatic activity (Figure 2(c)). Since the lower intracellular Fe^2+^ levels under 100 *μ*M NDGA exposure were the same as those observed for 50 *μ*M NDGA exposure, the bacteriostatic activity and the repression of intracellular Fe^2+^ levels are caused by different mechanisms.

The present study showed that NDGA repressed the SodB activity without bactericidal activity by itself (Figure 2). As a result, NDGA enhanced the bactericidal activity by ROS exposure (Figure 4). These results suggested that NDGA is effective for selective eradication therapy of pathogenic bacteria of inducing inflammatory response without affecting intestinal microbiota that does not induce an inflammatory response. Therefore,* H. pylori* eradication therapy with NDGA is expected to contribute to the development of selective eradication therapy of* H. pylori* by excessive ROS in host gastric mucosa without affecting intestinal microbiota.* H. pylori *eradication therapy often induces side effects such as diarrhea and soft stools. It was reported that 10–30% of ulcer patients receiving eradication therapy experienced diarrhea and soft stools [13, 14]. The reason for these side effects was assumed to be a disturbance in the composition of intestinal microbiota by antibiotics. Therefore,* H. pylori* eradication therapy with NDGA is expected to be effective in reducing the side effects such as diarrhea and soft stools.

Recently, the prevalence of Mtz resistance has increased in Asia and Europe [15–17]. It is suggested that the widespread use of Mtz may contribute to the increasing prevalence of Mtz resistance [16, 18]. Mtz is a prodrug, and its bactericidal activity is dependent on the generation of superoxide radicals mediated by the reduction of its nitro group to nitro anion radicals [19, 20]. Therefore, enhanced SodB activity is associated with the development of Mtz resistance [10]. In the present study, it was shown that NDGA repressed the SodB activity and then increased Mtz sensitivity in Mtz-resistant strains with SodB overexpression (Figure 3(b) and Table 1). From these results, it is expected that Mtz and NDGA combination therapy is effective for eradicating Mtz-resistant strains and preventing the development of Mtz resistance.

NDGA is the main metabolite of the creosote bush,* Larrea tridentata*, which is commonly known as chaparral or greasewood in the United States and as gobernadora or hediondilla in Mexico [21]. The creosote bush has been widely used in Mexican traditional herbal medicine [22]. It is known that NDGA is a potent scavenger of ROS such as peroxynitrite (ONOO^−^), singlet oxygen (^1^O_2_), hydroxyl radicals (^•^OH), superoxide anion (O_2_^−•^), H_2_O_2_, and hypochlorous acid (HOCl) [23]. Because the H_2_O_2_ sensitivity of* H. pylori *exposed to NDGA was significantly increased, it was thought that the SodB-inactivation caused by NDGA is independent of its ROS-scavenging activity. The present study is the first demonstration that NDGA suppresses the SodB activity of* H. pylori* via repression of intracellular Fe^2+^ by FecA1* in vitro*. Recent studies have indicated the inhibitory effect of NDGA against* N*-methyl-*N*-nitrosourea-initiated and* H. pylori*-promoted gastric carcinogenesis in Mongolian gerbils [24]. This inhibitory effect of NDGA might be associated with antioxidant activity and inhibitory effects on the progression of gastritis [24]. Therefore, it is expected that NDGA might be effective for both the eradication of* H. pylori* and the prevention of gastric carcinogenesis.

From our* in vitro* findings, it is expected that NDGA would bind to FecA1* in vivo*. To investigate whether NDGA binds to FecA1* in vivo*, further experiments in which fluorescence intensity and fluorescence localization are examined by immunohistochemical analysis using* H. pylori* infection Mongolian gerbil treated with fluorescently labeled NDGA are needed.

The reported acute NDGA LD_50_ ranges between 800 and 500 mg/kg body weight orally in rodents [22]. Although administration of 3% NDGA results in renal toxicity in rats, 0.5% and 1% NDGA did not exhibit toxicity [22]. Further studies are needed to determine the effect and toxicity of 50 *μ*M NDGA* in vivo*. In addition, it is important to analyze the binding mode of NDGA with FecA1* in vitro*, which would contribute to the chemical modification of NDGA that mediates the toxicity.

In conclusion, NDGA repressed the SodB activity associated with the gastric mucosal-colonization ability via repression of intracellular Fe^2+^ by FecA1 and increased the H_2_O_2_ sensitivity and the Mtz sensitivity of* H. pylori in vitro*.

## Figures and Tables

**Table 1:** Effect of NDGA on MICs (*μ*g/mL) of Mtz-resistant strains.

Strain numbers	Without NDGA	With 50 *μ*M NDGA
ATCC700392	32	4
pHel3::*sodB *		
KS0048	32	8
KS0145	128	16
